# (2*E*)-3-(2-Anthracen-2-yl)-1-(2-hy­droxy­phen­yl)prop-2-en-1-one

**DOI:** 10.1107/S1600536811007598

**Published:** 2011-03-05

**Authors:** Jerry P. Jasinski, Ray J. Butcher, V. Musthafa Khaleel, B. K. Sarojini, H. S. Yathirajan

**Affiliations:** a Department of Chemistry, Keene State College, 229 Main Street, Keene, NH 03435-2001, USA; b Department of Chemistry, Howard University, 525 College Street NW, Washington, DC 20059, USA; c Department of Chemistry, P.A. College of Engineering, Mangalore, 574 153, India; d Department of Studies in Chemistry, University of Mysore, Manasagangotri, Mysore 570 006, India

## Abstract

The asymmetric unit of the title compound, C_23_H_16_O_2_, contains two independent mol­ecules in which the dihedral angles between the anthracene ring system and the benzene ring are 73.0 (3) and 73.3 (3)°. In both independent mol­ecules, the hy­droxy group is involved in an intra­molecular O—H⋯O hydrogen bond. The crystal packing is stabilized by π–π inter­actions [centroid–centroid distances = 3.6518 (9), 3.7070 (9) and 3.7632 (9) Å] and weak inter­molecular C—H⋯O hydrogen bonds.

## Related literature

For related structures, see: Chantrapromma *et al.* (2009 ▶); Jasinski *et al.* (2010 ▶, 2011*a* ▶,*b* ▶); Lu *et al.* (2009 ▶); Suwunwong *et al.* (2009 ▶); Wang *et al.* (2009 ▶, 2010 ▶).
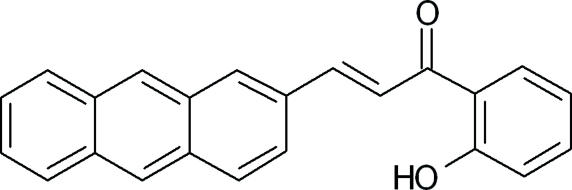


## Experimental

### 

#### Crystal data

C_23_H_16_O_2_
*M* *_r_* = 324.36
Monoclinic, 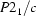

*a* = 14.0748 (5) Å
*b* = 13.7362 (5) Å
*c* = 16.9800 (8) Å
β = 101.487 (5)°
*V* = 3217.1 (2) Å^3^
*Z* = 8
Cu *K*α radiation
μ = 0.67 mm^−1^
*T* = 110 K
0.46 × 0.35 × 0.16 mm

#### Data collection

Oxford Diffraction Xcalibur diffractometer with a Ruby (Gemini Cu) detector
Absorption correction: multi-scan (*CrysAlis RED*; Oxford Diffraction, 2007 ▶) *T*_min_ = 0.530, *T*_max_ = 1.000
14048 measured reflections
6371 independent reflections
5277 reflections with *I* > 2σ(*I*)
*R*_int_ = 0.022

#### Refinement

*R*[*F* ^2^ > 2σ(*F* ^2^)] = 0.046
*wR*(*F* ^2^) = 0.134
*S* = 1.06
6371 reflections
453 parameters
H-atom parameters constrained
Δρ_max_ = 0.30 e Å^−3^
Δρ_min_ = −0.24 e Å^−3^

### 

Data collection: *CrysAlis PRO* (Oxford Diffraction, 2007 ▶); cell refinement: *CrysAlis PRO*; data reduction: *CrysAlis RED* (Oxford Diffraction, 2007 ▶); program(s) used to solve structure: *SHELXS97* (Sheldrick, 2008 ▶); program(s) used to refine structure: *SHELXL97* (Sheldrick, 2008 ▶); molecular graphics: *SHELXTL* (Sheldrick, 2008 ▶); software used to prepare material for publication: *SHELXTL*.

## Supplementary Material

Crystal structure: contains datablocks global, I. DOI: 10.1107/S1600536811007598/cv5048sup1.cif

Structure factors: contains datablocks I. DOI: 10.1107/S1600536811007598/cv5048Isup2.hkl

Additional supplementary materials:  crystallographic information; 3D view; checkCIF report

## Figures and Tables

**Table 1:** Hydrogen-bond geometry (Å, °)

*D*—H⋯*A*	*D*—H	H⋯*A*	*D*⋯*A*	*D*—H⋯*A*
O1*A*—H1*A*⋯O2*A*	0.84	1.83	2.5729 (15)	146
O1*B*—H1*B*⋯O2*B*	0.84	1.80	2.5452 (16)	146
C14*B*—H14*B*⋯O1*B*^i^	0.95	2.60	3.537 (2)	169

Symmetry code: (i) 
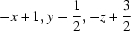
.

**Fig. 1.**
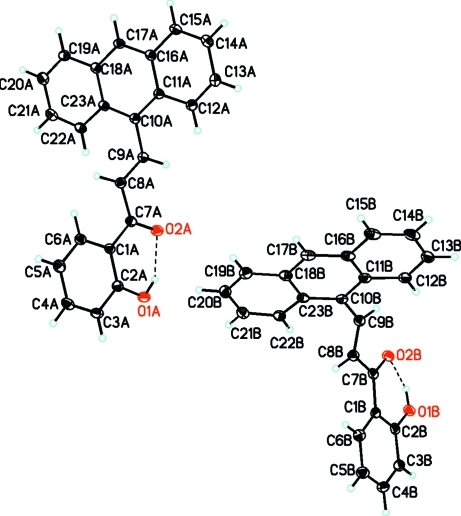
Two independent molecules of (I) showing the atom labeling scheme and 50% probability displacement ellipsoids. Dashed lines indicate intramolecular O—H···O hydrogen bonds.

**Fig. 2.**
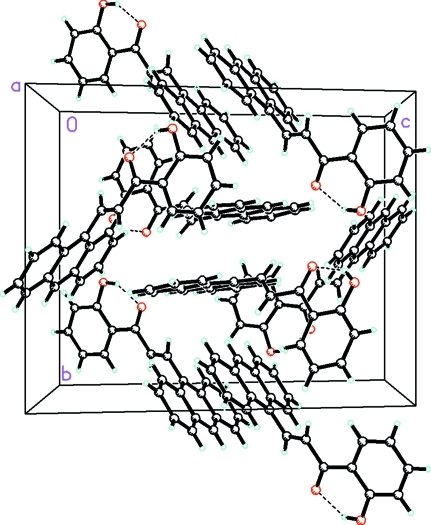
Packing diagram of the title compound viewed down the a axis. Dashed lines indicate intramolecular O—H···O hydrogen bonds.

**Fig. 3.**
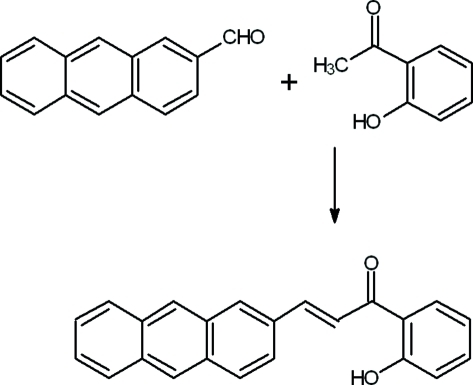
Reaction scheme for (I).

## supplementary crystallographic information

### Comment

In continuation to our studies on crystal structures of chalcones (Jasinski *et al.* 2010, 2011*a*, *b*) we report here the synthesis and crystal structure of the title compound, (I).

The asymmetric unit of (I) contains two independent molecules, A & B, respectively (Fig. 1). The dihedral angles between the mean planes of the 2-anthryl and benzene rings are 73.0 (9)° and 73.3 (3)° in A and B, respectively. Bond lengths and angles are normal and correspond to those observed in the related compounds ((*Z*)-3-(9-anthryl)-1-(4-methoxyphenyl) prop-2-en-1-one (Chantrapromma *et al.*, 2009), (*E*)-3-(anthracen-9-yl)-1-(4-bromophenyl)prop-2-en-1-one (Suwunwong *et al.*, 2009), (*Z*)-3-(9-anthryl)-1-(4-bromophenyl)-2-(4-nitro-1*H*-imidazol-1-yl) prop-2-en-1-one (Lu *et al.*, 2009), (*Z*)-3-(9-anthryl)-2-(4-nitro-1*H*-imidazol-1-yl)-1-*p*-\ tolylprop-2-en-1-one (Wang *et al.*, 2009) and (*E*)-3-(9-anthryl)-1-(4-fluorophenyl)-2-(4-nitro-1*H*-imidazol-1-\ yl) prop-2-en-1-one (Wang *et al.*, 2010).

Crystal packing (Fig. 2) is stabilized by π–π stacking interactions (Table 1) and weak intramolecular C—H···O hydrogen bonds (Table 2).

### Experimental

2-Hydroxyacetophenone (1.36 g, 0.01 mol) was mixed with 2- anthraldehyde (2.06 g, 0.01 mol) and dissolved in ethanol (40 ml). To this solution, 5 ml of KOH (50%) was added at 278 K. The reaction mixture stirred for 6 h and poured on to crushed ice (Fig. 3). The pH of this mixture was adjusted to 3–4 with 2 *M* HCl aqueous solution. The resulting crude yellow solid was filtered, washed successively with dilute HCl solution and distilled water and finally recrystallized from ethanol (95%) to give the pure chalcone. Crystals suitable for *x*-ray diffraction studies were grown by the slow evaporation of the solution of the compound in ethyl alcohol (m.p.: 393 K). Composition: Found (Calculated) for C_23_H_16_O_2_, C: 85.09 (85.16); H: 4.95 (4.97).

### Refinement

Atoms H1A and H1B were located on a Fourier map, and placed in idealized positions with O—H  0.84 Å. C-bound H atoms were placed in calculated positions (C—-H  0.95Å). All H atoms were refined as riding,  with U_iso_(H) = 1.2 *U*_eq_ of the parent atom.

### Crystal data

**Table:**

C_23_H_16_O_2_	*F*(000) = 1360
*M**_r_* = 324.36	*D*_x_ = 1.339 Mg m^−^^3^
Monoclinic, *P*2_1_/*c*	Cu *K*α radiation, λ = 1.54184 Å
Hall symbol: -P 2ybc	Cell parameters from 7472 reflections
*a* = 14.0748 (5) Å	θ = 4.5–74.2°
*b* = 13.7362 (5) Å	µ = 0.67 mm^−^^1^
*c* = 16.9800 (8) Å	*T* = 110 K
β = 101.487 (5)°	Plate, pale yellow
*V* = 3217.1 (2) Å^3^	0.46 × 0.35 × 0.16 mm
*Z* = 8	

### Data collection

**Table:**

Oxford Diffraction Xcalibur diffractometer with a Ruby (Gemini Cu) detector	6371 independent reflections
Radiation source: Enhance (Cu) X-ray Source	5277 reflections with *I* > 2σ(*I*)
graphite	*R*_int_ = 0.022
Detector resolution: 10.5081 pixels mm^-1^	θ_max_ = 74.3°, θ_min_ = 4.5°
ω scans	*h* = −17→17
Absorption correction: multi-scan (*CrysAlis RED*; Oxford Diffraction, 2007)	*k* = −13→16
*T*_min_ = 0.530, *T*_max_ = 1.000	*l* = −21→17
14048 measured reflections	

### Refinement

**Table:**

Refinement on *F*^2^	Primary atom site location: structure-invariant direct methods
Least-squares matrix: full	Secondary atom site location: difference Fourier map
*R*[*F*^2^ > 2σ(*F*^2^)] = 0.046	Hydrogen site location: inferred from neighbouring sites
*wR*(*F*^2^) = 0.134	H-atom parameters constrained
*S* = 1.06	*w* = 1/[σ^2^(*F*_o_^2^) + (0.082*P*)^2^ + 0.651*P*] where *P* = (*F*_o_^2^ + 2*F*_c_^2^)/3
6371 reflections	(Δ/σ)_max_ = 0.001
453 parameters	Δρ_max_ = 0.30 e Å^−^^3^
0 restraints	Δρ_min_ = −0.24 e Å^−^^3^

### Special details

**Table:**

Geometry. All e.s.d.'s (except the e.s.d. in the dihedral angle between two l.s. planes) are estimated using the full covariance matrix. The cell e.s.d.'s are taken into account individually in the estimation of e.s.d.'s in distances, angles and torsion angles; correlations between e.s.d.'s in cell parameters are only used when they are defined by crystal symmetry. An approximate (isotropic) treatment of cell e.s.d.'s is used for estimating e.s.d.'s involving l.s. planes.
Refinement. Refinement of *F*^2^ against ALL reflections. The weighted *R*-factor *wR* and goodness of fit *S* are based on *F*^2^, conventional *R*-factors *R* are based on *F*, with *F* set to zero for negative *F*^2^. The threshold expression of *F*^2^ > σ(*F*^2^) is used only for calculating *R*-factors(gt) *etc*. and is not relevant to the choice of reflections for refinement. *R*-factors based on *F*^2^ are statistically about twice as large as those based on *F*, and *R*- factors based on ALL data will be even larger.

### Fractional atomic coordinates and isotropic or equivalent isotropic displacement parameters (Å^2^)

**Table:**

	*x*	*y*	*z*	*U*_iso_*/*U*_eq_	
O1A	0.96585 (8)	0.60784 (8)	0.83860 (7)	0.0324 (3)	
H1A	0.9210	0.5710	0.8160	0.039*	
O2A	0.81576 (8)	0.55812 (8)	0.73334 (7)	0.0331 (3)	
C1A	0.88430 (10)	0.71509 (11)	0.73290 (8)	0.0240 (3)	
C2A	0.95647 (10)	0.69567 (11)	0.80224 (9)	0.0261 (3)	
C3A	1.02217 (10)	0.76822 (13)	0.83456 (9)	0.0303 (3)	
H3AA	1.0707	0.7547	0.8807	0.036*	
C4A	1.01718 (11)	0.85966 (12)	0.79998 (10)	0.0319 (3)	
H4AA	1.0621	0.9086	0.8226	0.038*	
C5A	0.94648 (11)	0.88039 (12)	0.73208 (10)	0.0312 (3)	
H5AA	0.9429	0.9434	0.7085	0.037*	
C6A	0.88169 (10)	0.80891 (11)	0.69930 (9)	0.0264 (3)	
H6AA	0.8340	0.8234	0.6528	0.032*	
C7A	0.81576 (10)	0.63764 (11)	0.69853 (9)	0.0255 (3)	
C8A	0.74674 (11)	0.65488 (11)	0.62179 (9)	0.0262 (3)	
H8AA	0.7582	0.7072	0.5883	0.031*	
C9A	0.66916 (10)	0.59885 (10)	0.59865 (9)	0.0244 (3)	
H9AA	0.6597	0.5466	0.6329	0.029*	
C10A	0.59645 (10)	0.61196 (10)	0.52339 (9)	0.0233 (3)	
C11A	0.49737 (10)	0.62045 (10)	0.52774 (9)	0.0230 (3)	
C12A	0.46396 (11)	0.61652 (11)	0.60210 (9)	0.0273 (3)	
H12A	0.5097	0.6073	0.6508	0.033*	
C13A	0.36839 (12)	0.62563 (11)	0.60448 (10)	0.0300 (3)	
H13A	0.3484	0.6221	0.6546	0.036*	
C14A	0.29797 (11)	0.64039 (12)	0.53282 (10)	0.0309 (3)	
H14A	0.2314	0.6467	0.5354	0.037*	
C15A	0.32575 (11)	0.64553 (11)	0.46082 (10)	0.0278 (3)	
H15A	0.2783	0.6555	0.4132	0.033*	
C16A	0.42598 (10)	0.63611 (10)	0.45561 (9)	0.0242 (3)	
C17A	0.45523 (11)	0.64314 (10)	0.38216 (9)	0.0251 (3)	
H17A	0.4078	0.6535	0.3346	0.030*	
C18A	0.55268 (11)	0.63533 (10)	0.37680 (9)	0.0247 (3)	
C19A	0.58188 (12)	0.64459 (12)	0.30104 (9)	0.0307 (3)	
H19A	0.5344	0.6577	0.2541	0.037*	
C20A	0.67614 (12)	0.63502 (13)	0.29520 (10)	0.0342 (4)	
H20A	0.6944	0.6427	0.2446	0.041*	
C21A	0.74762 (11)	0.61345 (12)	0.36476 (10)	0.0320 (4)	
H21A	0.8133	0.6051	0.3601	0.038*	
C22A	0.72298 (11)	0.60470 (11)	0.43796 (9)	0.0276 (3)	
H22A	0.7719	0.5897	0.4835	0.033*	
C23A	0.62502 (10)	0.61761 (10)	0.44803 (9)	0.0239 (3)	
O1B	0.87649 (8)	0.37852 (9)	0.86203 (7)	0.0357 (3)	
H1B	0.8338	0.3737	0.8199	0.043*	
O2B	0.76730 (8)	0.29254 (8)	0.74519 (7)	0.0326 (3)	
C1B	0.88557 (10)	0.20563 (12)	0.83681 (9)	0.0264 (3)	
C2B	0.91401 (11)	0.29000 (12)	0.88336 (9)	0.0296 (3)	
C3B	0.98406 (11)	0.28300 (14)	0.95397 (10)	0.0348 (4)	
H3BA	1.0015	0.3390	0.9864	0.042*	
C4B	1.02796 (12)	0.19501 (14)	0.97663 (10)	0.0363 (4)	
H4BA	1.0762	0.1912	1.0244	0.044*	
C5B	1.00302 (12)	0.11175 (13)	0.93085 (10)	0.0344 (4)	
H5BA	1.0347	0.0517	0.9466	0.041*	
C6B	0.93192 (11)	0.11713 (12)	0.86238 (10)	0.0302 (3)	
H6BA	0.9138	0.0598	0.8317	0.036*	
C7B	0.80797 (10)	0.21316 (12)	0.76505 (9)	0.0271 (3)	
C8B	0.77549 (11)	0.12518 (12)	0.71763 (9)	0.0296 (3)	
H8BA	0.8164	0.0696	0.7222	0.035*	
C9B	0.68909 (11)	0.12307 (12)	0.66833 (9)	0.0276 (3)	
H9BA	0.6512	0.1808	0.6635	0.033*	
C10B	0.64816 (10)	0.03859 (11)	0.62117 (8)	0.0245 (3)	
C11B	0.55384 (10)	0.00678 (11)	0.62741 (8)	0.0261 (3)	
C12B	0.49770 (11)	0.05492 (13)	0.67701 (9)	0.0327 (4)	
H12B	0.5231	0.1110	0.7067	0.039*	
C13B	0.40761 (12)	0.02110 (15)	0.68231 (10)	0.0391 (4)	
H13B	0.3709	0.0546	0.7151	0.047*	
C14B	0.36860 (11)	−0.06279 (15)	0.63980 (10)	0.0397 (4)	
H14B	0.3064	−0.0858	0.6448	0.048*	
C15B	0.41931 (12)	−0.11068 (13)	0.59188 (10)	0.0344 (4)	
H15B	0.3920	−0.1667	0.5632	0.041*	
C16B	0.51345 (11)	−0.07794 (12)	0.58384 (9)	0.0278 (3)	
C17B	0.56580 (11)	−0.12588 (11)	0.53403 (9)	0.0285 (3)	
H17B	0.5385	−0.1817	0.5050	0.034*	
C18B	0.65734 (11)	−0.09390 (11)	0.52567 (9)	0.0261 (3)	
C19B	0.70820 (12)	−0.14151 (11)	0.47156 (10)	0.0309 (3)	
H19B	0.6799	−0.1965	0.4420	0.037*	
C20B	0.79625 (12)	−0.10940 (12)	0.46176 (10)	0.0327 (3)	
H20B	0.8294	−0.1422	0.4260	0.039*	
C21B	0.83880 (11)	−0.02683 (12)	0.50492 (9)	0.0312 (3)	
H21B	0.9003	−0.0045	0.4974	0.037*	
C22B	0.79310 (11)	0.02095 (11)	0.55691 (9)	0.0269 (3)	
H22B	0.8230	0.0763	0.5849	0.032*	
C23B	0.70031 (10)	−0.01093 (11)	0.57023 (8)	0.0241 (3)	

### Atomic displacement parameters (Å^2^)

**Table:**

	*U*^11^	*U*^22^	*U*^33^	*U*^12^	*U*^13^	*U*^23^
O1A	0.0283 (6)	0.0336 (6)	0.0318 (6)	−0.0002 (4)	−0.0021 (4)	0.0019 (5)
O2A	0.0317 (6)	0.0280 (6)	0.0355 (6)	−0.0025 (4)	−0.0032 (5)	0.0049 (5)
C1A	0.0196 (6)	0.0293 (7)	0.0236 (6)	0.0005 (5)	0.0057 (5)	−0.0032 (6)
C2A	0.0222 (7)	0.0322 (8)	0.0250 (7)	0.0026 (6)	0.0073 (6)	−0.0021 (6)
C3A	0.0217 (7)	0.0432 (9)	0.0252 (7)	0.0005 (6)	0.0023 (6)	−0.0063 (6)
C4A	0.0258 (7)	0.0364 (9)	0.0338 (8)	−0.0072 (6)	0.0066 (6)	−0.0118 (7)
C5A	0.0299 (8)	0.0297 (8)	0.0346 (8)	−0.0020 (6)	0.0081 (6)	−0.0036 (6)
C6A	0.0228 (7)	0.0312 (8)	0.0253 (7)	0.0003 (6)	0.0047 (5)	−0.0023 (6)
C7A	0.0225 (7)	0.0264 (7)	0.0273 (7)	0.0015 (6)	0.0041 (6)	−0.0010 (6)
C8A	0.0258 (7)	0.0246 (7)	0.0271 (7)	−0.0002 (6)	0.0025 (6)	0.0008 (6)
C9A	0.0252 (7)	0.0213 (7)	0.0261 (7)	0.0006 (5)	0.0038 (6)	−0.0004 (6)
C10A	0.0240 (7)	0.0163 (6)	0.0285 (7)	−0.0019 (5)	0.0024 (6)	−0.0017 (5)
C11A	0.0244 (7)	0.0168 (6)	0.0270 (7)	−0.0029 (5)	0.0029 (6)	−0.0013 (5)
C12A	0.0287 (7)	0.0240 (7)	0.0280 (7)	−0.0016 (6)	0.0030 (6)	0.0003 (6)
C13A	0.0320 (8)	0.0285 (8)	0.0314 (8)	−0.0018 (6)	0.0107 (6)	−0.0003 (6)
C14A	0.0237 (7)	0.0294 (8)	0.0403 (8)	−0.0007 (6)	0.0075 (6)	−0.0012 (7)
C15A	0.0223 (7)	0.0252 (7)	0.0339 (8)	−0.0007 (6)	0.0012 (6)	−0.0011 (6)
C16A	0.0227 (7)	0.0178 (6)	0.0309 (7)	−0.0021 (5)	0.0027 (6)	−0.0020 (5)
C17A	0.0245 (7)	0.0212 (7)	0.0267 (7)	−0.0017 (5)	−0.0018 (6)	−0.0021 (6)
C18A	0.0263 (7)	0.0204 (7)	0.0266 (7)	−0.0037 (5)	0.0035 (6)	−0.0047 (5)
C19A	0.0312 (8)	0.0327 (8)	0.0263 (7)	−0.0053 (6)	0.0016 (6)	−0.0043 (6)
C20A	0.0347 (8)	0.0413 (9)	0.0281 (8)	−0.0080 (7)	0.0102 (7)	−0.0084 (7)
C21A	0.0250 (7)	0.0342 (8)	0.0373 (8)	−0.0043 (6)	0.0077 (6)	−0.0106 (7)
C22A	0.0244 (7)	0.0250 (7)	0.0321 (7)	−0.0023 (6)	0.0024 (6)	−0.0063 (6)
C23A	0.0231 (7)	0.0189 (7)	0.0286 (7)	−0.0033 (5)	0.0024 (6)	−0.0044 (5)
O1B	0.0278 (6)	0.0360 (6)	0.0401 (6)	0.0039 (5)	−0.0006 (5)	−0.0118 (5)
O2B	0.0275 (5)	0.0310 (6)	0.0367 (6)	0.0024 (4)	0.0001 (5)	−0.0045 (5)
C1B	0.0210 (7)	0.0344 (8)	0.0246 (7)	−0.0022 (6)	0.0063 (6)	−0.0017 (6)
C2B	0.0220 (7)	0.0361 (8)	0.0320 (8)	−0.0001 (6)	0.0088 (6)	−0.0051 (7)
C3B	0.0271 (8)	0.0438 (9)	0.0318 (8)	−0.0018 (7)	0.0021 (6)	−0.0114 (7)
C4B	0.0279 (8)	0.0532 (11)	0.0264 (7)	0.0004 (7)	0.0016 (6)	−0.0002 (7)
C5B	0.0316 (8)	0.0383 (9)	0.0325 (8)	0.0007 (7)	0.0046 (7)	0.0076 (7)
C6B	0.0295 (8)	0.0302 (8)	0.0310 (7)	−0.0039 (6)	0.0062 (6)	0.0011 (6)
C7B	0.0216 (7)	0.0310 (8)	0.0295 (7)	−0.0001 (6)	0.0070 (6)	−0.0009 (6)
C8B	0.0275 (7)	0.0296 (8)	0.0305 (7)	0.0011 (6)	0.0031 (6)	−0.0030 (6)
C9B	0.0248 (7)	0.0291 (8)	0.0287 (7)	−0.0007 (6)	0.0049 (6)	−0.0011 (6)
C10B	0.0222 (7)	0.0263 (7)	0.0228 (6)	−0.0013 (5)	−0.0008 (5)	0.0040 (6)
C11B	0.0221 (7)	0.0307 (8)	0.0233 (7)	−0.0005 (6)	−0.0007 (5)	0.0074 (6)
C12B	0.0256 (7)	0.0459 (10)	0.0249 (7)	0.0004 (7)	0.0004 (6)	0.0030 (7)
C13B	0.0252 (8)	0.0647 (12)	0.0275 (8)	0.0051 (8)	0.0050 (6)	0.0101 (8)
C14B	0.0209 (7)	0.0589 (12)	0.0373 (9)	−0.0066 (7)	0.0010 (7)	0.0189 (8)
C15B	0.0261 (8)	0.0360 (9)	0.0374 (8)	−0.0083 (6)	−0.0023 (6)	0.0133 (7)
C16B	0.0236 (7)	0.0281 (8)	0.0286 (7)	−0.0039 (6)	−0.0020 (6)	0.0107 (6)
C17B	0.0288 (8)	0.0212 (7)	0.0311 (7)	−0.0046 (6)	−0.0044 (6)	0.0053 (6)
C18B	0.0269 (7)	0.0205 (7)	0.0284 (7)	0.0007 (6)	−0.0007 (6)	0.0053 (6)
C19B	0.0363 (8)	0.0219 (7)	0.0316 (8)	0.0030 (6)	0.0000 (6)	0.0004 (6)
C20B	0.0349 (8)	0.0305 (8)	0.0328 (8)	0.0075 (7)	0.0071 (7)	0.0002 (6)
C21B	0.0264 (7)	0.0335 (8)	0.0341 (8)	0.0011 (6)	0.0065 (6)	0.0051 (7)
C22B	0.0241 (7)	0.0254 (7)	0.0298 (7)	−0.0030 (6)	0.0021 (6)	0.0021 (6)
C23B	0.0229 (7)	0.0225 (7)	0.0247 (7)	0.0002 (5)	−0.0002 (6)	0.0051 (6)

### Geometric parameters (Å, °)

**Table:**

O1A—C2A	1.3498 (19)	O1B—C2B	1.346 (2)
O1A—H1A	0.8400	O1B—H1B	0.8400
O2A—C7A	1.2420 (19)	O2B—C7B	1.2464 (19)
C1A—C6A	1.407 (2)	C1B—C6B	1.407 (2)
C1A—C2A	1.419 (2)	C1B—C2B	1.415 (2)
C1A—C7A	1.476 (2)	C1B—C7B	1.469 (2)
C2A—C3A	1.395 (2)	C2B—C3B	1.396 (2)
C3A—C4A	1.382 (2)	C3B—C4B	1.377 (3)
C3A—H3AA	0.9500	C3B—H3BA	0.9500
C4A—C5A	1.394 (2)	C4B—C5B	1.388 (3)
C4A—H4AA	0.9500	C4B—H4BA	0.9500
C5A—C6A	1.380 (2)	C5B—C6B	1.377 (2)
C5A—H5AA	0.9500	C5B—H5BA	0.9500
C6A—H6AA	0.9500	C6B—H6BA	0.9500
C7A—C8A	1.481 (2)	C7B—C8B	1.473 (2)
C8A—C9A	1.330 (2)	C8B—C9B	1.332 (2)
C8A—H8AA	0.9500	C8B—H8BA	0.9500
C9A—C10A	1.481 (2)	C9B—C10B	1.461 (2)
C9A—H9AA	0.9500	C9B—H9BA	0.9500
C10A—C11A	1.416 (2)	C10B—C23B	1.415 (2)
C10A—C23A	1.418 (2)	C10B—C11B	1.421 (2)
C11A—C12A	1.433 (2)	C11B—C12B	1.427 (2)
C11A—C16A	1.437 (2)	C11B—C16B	1.434 (2)
C12A—C13A	1.360 (2)	C12B—C13B	1.370 (2)
C12A—H12A	0.9500	C12B—H12B	0.9500
C13A—C14A	1.423 (2)	C13B—C14B	1.412 (3)
C13A—H13A	0.9500	C13B—H13B	0.9500
C14A—C15A	1.358 (2)	C14B—C15B	1.355 (3)
C14A—H14A	0.9500	C14B—H14B	0.9500
C15A—C16A	1.437 (2)	C15B—C16B	1.431 (2)
C15A—H15A	0.9500	C15B—H15B	0.9500
C16A—C17A	1.392 (2)	C16B—C17B	1.393 (2)
C17A—C18A	1.397 (2)	C17B—C18B	1.395 (2)
C17A—H17A	0.9500	C17B—H17B	0.9500
C18A—C19A	1.432 (2)	C18B—C19B	1.430 (2)
C18A—C23A	1.438 (2)	C18B—C23B	1.434 (2)
C19A—C20A	1.356 (2)	C19B—C20B	1.357 (2)
C19A—H19A	0.9500	C19B—H19B	0.9500
C20A—C21A	1.422 (2)	C20B—C21B	1.417 (2)
C20A—H20A	0.9500	C20B—H20B	0.9500
C21A—C22A	1.361 (2)	C21B—C22B	1.360 (2)
C21A—H21A	0.9500	C21B—H21B	0.9500
C22A—C23A	1.434 (2)	C22B—C23B	1.437 (2)
C22A—H22A	0.9500	C22B—H22B	0.9500
			
Cg1···Cg4^i^	3.652 (2)	Cg5···Cg5^iii^	3.763 (2)
Cg2···Cg3^ii^	3.707 (2)		
			
C2A—O1A—H1A	109.5	C2B—O1B—H1B	109.5
C6A—C1A—C2A	117.73 (14)	C6B—C1B—C2B	118.24 (14)
C6A—C1A—C7A	122.52 (13)	C6B—C1B—C7B	122.64 (14)
C2A—C1A—C7A	119.75 (14)	C2B—C1B—C7B	119.11 (14)
O1A—C2A—C3A	117.49 (13)	O1B—C2B—C3B	117.74 (15)
O1A—C2A—C1A	122.44 (14)	O1B—C2B—C1B	122.51 (14)
C3A—C2A—C1A	120.06 (14)	C3B—C2B—C1B	119.75 (15)
C4A—C3A—C2A	120.55 (14)	C4B—C3B—C2B	120.10 (16)
C4A—C3A—H3AA	119.7	C4B—C3B—H3BA	119.9
C2A—C3A—H3AA	119.7	C2B—C3B—H3BA	119.9
C3A—C4A—C5A	120.29 (14)	C3B—C4B—C5B	121.15 (15)
C3A—C4A—H4AA	119.9	C3B—C4B—H4BA	119.4
C5A—C4A—H4AA	119.9	C5B—C4B—H4BA	119.4
C6A—C5A—C4A	119.60 (15)	C6B—C5B—C4B	119.27 (16)
C6A—C5A—H5AA	120.2	C6B—C5B—H5BA	120.4
C4A—C5A—H5AA	120.2	C4B—C5B—H5BA	120.4
C5A—C6A—C1A	121.77 (14)	C5B—C6B—C1B	121.44 (15)
C5A—C6A—H6AA	119.1	C5B—C6B—H6BA	119.3
C1A—C6A—H6AA	119.1	C1B—C6B—H6BA	119.3
O2A—C7A—C1A	120.50 (13)	O2B—C7B—C1B	120.76 (14)
O2A—C7A—C8A	119.82 (14)	O2B—C7B—C8B	119.60 (13)
C1A—C7A—C8A	119.68 (13)	C1B—C7B—C8B	119.60 (14)
C9A—C8A—C7A	121.57 (14)	C9B—C8B—C7B	120.42 (14)
C9A—C8A—H8AA	119.2	C9B—C8B—H8BA	119.8
C7A—C8A—H8AA	119.2	C7B—C8B—H8BA	119.8
C8A—C9A—C10A	124.78 (14)	C8B—C9B—C10B	124.92 (15)
C8A—C9A—H9AA	117.6	C8B—C9B—H9BA	117.5
C10A—C9A—H9AA	117.6	C10B—C9B—H9BA	117.5
C11A—C10A—C23A	120.13 (13)	C23B—C10B—C11B	120.24 (14)
C11A—C10A—C9A	118.93 (13)	C23B—C10B—C9B	121.38 (13)
C23A—C10A—C9A	120.94 (13)	C11B—C10B—C9B	118.38 (13)
C10A—C11A—C12A	122.82 (13)	C10B—C11B—C12B	122.46 (15)
C10A—C11A—C16A	119.77 (13)	C10B—C11B—C16B	119.41 (14)
C12A—C11A—C16A	117.39 (13)	C12B—C11B—C16B	118.12 (14)
C13A—C12A—C11A	121.52 (14)	C13B—C12B—C11B	120.68 (17)
C13A—C12A—H12A	119.2	C13B—C12B—H12B	119.7
C11A—C12A—H12A	119.2	C11B—C12B—H12B	119.7
C12A—C13A—C14A	120.84 (14)	C12B—C13B—C14B	120.93 (17)
C12A—C13A—H13A	119.6	C12B—C13B—H13B	119.5
C14A—C13A—H13A	119.6	C14B—C13B—H13B	119.5
C15A—C14A—C13A	120.07 (14)	C15B—C14B—C13B	120.38 (15)
C15A—C14A—H14A	120.0	C15B—C14B—H14B	119.8
C13A—C14A—H14A	120.0	C13B—C14B—H14B	119.8
C14A—C15A—C16A	120.88 (14)	C14B—C15B—C16B	120.87 (16)
C14A—C15A—H15A	119.6	C14B—C15B—H15B	119.6
C16A—C15A—H15A	119.6	C16B—C15B—H15B	119.6
C17A—C16A—C15A	121.20 (14)	C17B—C16B—C15B	121.40 (15)
C17A—C16A—C11A	119.50 (13)	C17B—C16B—C11B	119.58 (14)
C15A—C16A—C11A	119.30 (14)	C15B—C16B—C11B	119.02 (15)
C16A—C17A—C18A	121.50 (13)	C16B—C17B—C18B	121.59 (14)
C16A—C17A—H17A	119.3	C16B—C17B—H17B	119.2
C18A—C17A—H17A	119.3	C18B—C17B—H17B	119.2
C17A—C18A—C19A	120.86 (14)	C17B—C18B—C19B	120.79 (14)
C17A—C18A—C23A	119.88 (13)	C17B—C18B—C23B	119.85 (14)
C19A—C18A—C23A	119.26 (13)	C19B—C18B—C23B	119.34 (14)
C20A—C19A—C18A	121.13 (15)	C20B—C19B—C18B	121.09 (15)
C20A—C19A—H19A	119.4	C20B—C19B—H19B	119.5
C18A—C19A—H19A	119.4	C18B—C19B—H19B	119.5
C19A—C20A—C21A	120.00 (15)	C19B—C20B—C21B	119.95 (15)
C19A—C20A—H20A	120.0	C19B—C20B—H20B	120.0
C21A—C20A—H20A	120.0	C21B—C20B—H20B	120.0
C22A—C21A—C20A	120.69 (14)	C22B—C21B—C20B	121.08 (15)
C22A—C21A—H21A	119.7	C22B—C21B—H21B	119.5
C20A—C21A—H21A	119.7	C20B—C21B—H21B	119.5
C21A—C22A—C23A	121.61 (14)	C21B—C22B—C23B	121.14 (14)
C21A—C22A—H22A	119.2	C21B—C22B—H22B	119.4
C23A—C22A—H22A	119.2	C23B—C22B—H22B	119.4
C10A—C23A—C22A	123.59 (14)	C10B—C23B—C18B	119.28 (13)
C10A—C23A—C18A	119.19 (13)	C10B—C23B—C22B	123.23 (14)
C22A—C23A—C18A	117.21 (13)	C18B—C23B—C22B	117.40 (13)
			
C6A—C1A—C2A—O1A	−179.30 (13)	C6B—C1B—C2B—O1B	177.39 (14)
C7A—C1A—C2A—O1A	0.4 (2)	C7B—C1B—C2B—O1B	−3.8 (2)
C6A—C1A—C2A—C3A	−0.4 (2)	C6B—C1B—C2B—C3B	−2.1 (2)
C7A—C1A—C2A—C3A	179.31 (13)	C7B—C1B—C2B—C3B	176.67 (13)
O1A—C2A—C3A—C4A	179.54 (13)	O1B—C2B—C3B—C4B	−177.06 (15)
C1A—C2A—C3A—C4A	0.6 (2)	C1B—C2B—C3B—C4B	2.5 (2)
C2A—C3A—C4A—C5A	−0.2 (2)	C2B—C3B—C4B—C5B	−0.8 (3)
C3A—C4A—C5A—C6A	−0.3 (2)	C3B—C4B—C5B—C6B	−1.2 (2)
C4A—C5A—C6A—C1A	0.5 (2)	C4B—C5B—C6B—C1B	1.5 (2)
C2A—C1A—C6A—C5A	−0.1 (2)	C2B—C1B—C6B—C5B	0.1 (2)
C7A—C1A—C6A—C5A	−179.83 (14)	C7B—C1B—C6B—C5B	−178.59 (14)
C6A—C1A—C7A—O2A	−174.60 (14)	C6B—C1B—C7B—O2B	178.57 (14)
C2A—C1A—C7A—O2A	5.7 (2)	C2B—C1B—C7B—O2B	−0.1 (2)
C6A—C1A—C7A—C8A	5.6 (2)	C6B—C1B—C7B—C8B	0.7 (2)
C2A—C1A—C7A—C8A	−174.06 (12)	C2B—C1B—C7B—C8B	−177.97 (13)
O2A—C7A—C8A—C9A	17.1 (2)	O2B—C7B—C8B—C9B	−17.9 (2)
C1A—C7A—C8A—C9A	−163.12 (14)	C1B—C7B—C8B—C9B	159.94 (14)
C7A—C8A—C9A—C10A	179.36 (13)	C7B—C8B—C9B—C10B	−177.36 (14)
C8A—C9A—C10A—C11A	−125.78 (16)	C8B—C9B—C10B—C23B	−52.8 (2)
C8A—C9A—C10A—C23A	53.2 (2)	C8B—C9B—C10B—C11B	127.28 (17)
C23A—C10A—C11A—C12A	−179.24 (13)	C23B—C10B—C11B—C12B	−179.11 (14)
C9A—C10A—C11A—C12A	−0.3 (2)	C9B—C10B—C11B—C12B	0.8 (2)
C23A—C10A—C11A—C16A	−0.8 (2)	C23B—C10B—C11B—C16B	2.2 (2)
C9A—C10A—C11A—C16A	178.16 (13)	C9B—C10B—C11B—C16B	−177.89 (13)
C10A—C11A—C12A—C13A	179.50 (14)	C10B—C11B—C12B—C13B	−179.02 (14)
C16A—C11A—C12A—C13A	1.0 (2)	C16B—C11B—C12B—C13B	−0.3 (2)
C11A—C12A—C13A—C14A	−0.6 (2)	C11B—C12B—C13B—C14B	0.8 (2)
C12A—C13A—C14A—C15A	0.0 (2)	C12B—C13B—C14B—C15B	−1.0 (3)
C13A—C14A—C15A—C16A	0.1 (2)	C13B—C14B—C15B—C16B	0.5 (2)
C14A—C15A—C16A—C17A	−178.77 (14)	C14B—C15B—C16B—C17B	−179.23 (15)
C14A—C15A—C16A—C11A	0.4 (2)	C14B—C15B—C16B—C11B	0.0 (2)
C10A—C11A—C16A—C17A	−0.3 (2)	C10B—C11B—C16B—C17B	−2.1 (2)
C12A—C11A—C16A—C17A	178.26 (13)	C12B—C11B—C16B—C17B	179.13 (14)
C10A—C11A—C16A—C15A	−179.43 (13)	C10B—C11B—C16B—C15B	178.64 (13)
C12A—C11A—C16A—C15A	−0.9 (2)	C12B—C11B—C16B—C15B	−0.1 (2)
C15A—C16A—C17A—C18A	179.15 (13)	C15B—C16B—C17B—C18B	179.63 (14)
C11A—C16A—C17A—C18A	0.0 (2)	C11B—C16B—C17B—C18B	0.4 (2)
C16A—C17A—C18A—C19A	−178.78 (14)	C16B—C17B—C18B—C19B	−177.25 (14)
C16A—C17A—C18A—C23A	1.3 (2)	C16B—C17B—C18B—C23B	1.2 (2)
C17A—C18A—C19A—C20A	−178.58 (15)	C17B—C18B—C19B—C20B	178.46 (14)
C23A—C18A—C19A—C20A	1.3 (2)	C23B—C18B—C19B—C20B	0.0 (2)
C18A—C19A—C20A—C21A	1.3 (3)	C18B—C19B—C20B—C21B	−0.6 (2)
C19A—C20A—C21A—C22A	−1.7 (3)	C19B—C20B—C21B—C22B	0.5 (2)
C20A—C21A—C22A—C23A	−0.6 (2)	C20B—C21B—C22B—C23B	0.3 (2)
C11A—C10A—C23A—C22A	−176.64 (13)	C11B—C10B—C23B—C18B	−0.6 (2)
C9A—C10A—C23A—C22A	4.4 (2)	C9B—C10B—C23B—C18B	179.51 (13)
C11A—C10A—C23A—C18A	2.1 (2)	C11B—C10B—C23B—C22B	175.76 (13)
C9A—C10A—C23A—C18A	−176.86 (13)	C9B—C10B—C23B—C22B	−4.2 (2)
C21A—C22A—C23A—C10A	−178.13 (14)	C17B—C18B—C23B—C10B	−1.1 (2)
C21A—C22A—C23A—C18A	3.1 (2)	C19B—C18B—C23B—C10B	177.35 (13)
C17A—C18A—C23A—C10A	−2.4 (2)	C17B—C18B—C23B—C22B	−177.69 (13)
C19A—C18A—C23A—C10A	177.75 (13)	C19B—C18B—C23B—C22B	0.8 (2)
C17A—C18A—C23A—C22A	176.46 (13)	C21B—C22B—C23B—C10B	−177.36 (14)
C19A—C18A—C23A—C22A	−3.4 (2)	C21B—C22B—C23B—C18B	−1.0 (2)

Symmetry codes: (i) −*x*+2, *y*+1/2, −*z*+3/2; (ii) −*x*+1, −*y*+1, −*z*+1; (iii) −*x*+1, −*y*, −*z*+1.

### Hydrogen-bond geometry (Å, °)

**Table:**

*D*—H···*A*	*D*—H	H···*A*	*D*···*A*	*D*—H···*A*
O1A—H1A···O2A	0.84	1.83	2.5729 (15)	146
O1B—H1B···O2B	0.84	1.80	2.5452 (16)	146
C14B—H14B···O1B^iv^	0.95	2.60	3.537 (2)	169

Symmetry codes: (iv) −*x*+1, *y*−1/2, −*z*+3/2.
